# Challenges Reconciling Theory and Experiments in the Prediction of Lattice Thermal Conductivity: The Case of Cu-Based Sulvanites

**DOI:** 10.1021/acs.chemmater.4c01343

**Published:** 2024-09-04

**Authors:** Irene Caro-Campos, Marta María González-Barrios, Oscar J. Dura, Erik Fransson, Jose J. Plata, David Ávila, Javier Fdez Sanz, Jesús Prado-Gonjal, Antonio M. Márquez

**Affiliations:** † Departamento de Química Física, Facultad de Química, Universidad de Sevilla, E-41012 Seville, Spain; ‡ Departamento de Química Inorgánica, Universidad Complutense de Madrid, E-28040 Madrid, Spain; § Departamento de Física Aplicada, Universidad de Castilla-La Mancha, E-13071 Ciudad Real, Spain; ∥ Department of Physics, Chalmers University of Technology, SE-41296 Gothenburg, Sweden

## Abstract

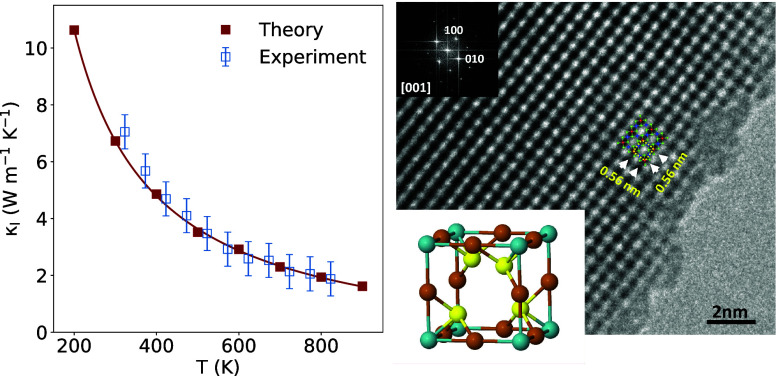

The exploration of large chemical spaces in search of new thermoelectric materials requires the integration of experiments, theory, simulations, and data science. The development of high-throughput strategies that combine DFT calculations with machine learning has emerged as a powerful approach to discovering new materials. However, experimental validation is crucial to confirm the accuracy of these workflows. This validation becomes especially important in understanding the transport properties that govern the thermoelectric performance of materials since they are highly influenced by synthetic, processing, and operating conditions. In this work, we explore the thermal conductivity of Cu-based sulvanites by using a combination of theoretical and experimental methods. Previous discrepancies and significant variations in reported data for Cu_3_VS_4_ and Cu_3_VSe_4_ are explained using the Boltzmann Transport Equation for phonons and by synthesizing well-characterized defect-free samples. The use of machine learning approaches for extracting high-order force constants opens doors to charting the lattice thermal conductivity across the entire Cu-based sulvanite family—finding not only materials with κ_*l*_ values below 2 W m^–1^ K^–1^ at moderate temperatures but also rationalizing their thermal transport properties based on chemical composition.

## Introduction

Computational Materials Science has emerged as a pillar in the discovery and prediction of solids. In addition to the development of simulations based on density functional theory, DFT, during the last century, its combination with artificial intelligence, AI, has established a new paradigm in science in the last 20 years.^1,2^ The synergy between both fields has resulted in not only more accurate but also more computational affordable methods which can be used to explore large chemical spaces.^3,4^ In this scenario, Computational Materials Science has reached a new level of maturity which not only complements and corroborates experimental findings but also leads in the search for, and prediction of, new materials.^5^ However, this progress has not been uniformly applied across all types of materials, properties, and applications. For instance, while the prediction of the optoelectronic properties for the discovery of new photovoltaic materials has achieved significant success,^6^ calculating transport properties for searching and optimizing thermoelectric, TE, materials can still be considered a challenge.^7−10^

The advance on computational-guided thermoelectric materials discovery^11^ is hampered by three main obstacles. First, thermoelectric and transport properties depend on the study of phonon and/or electron scattering which are computationally demanding and difficult to implement in a high-throughput fashion.^12^ For instance, predicting lattice thermal conductivity, κ_l_, requires the calculation of interatomic force constants, IFCs, and the solution of the Boltzmann transport equation, BTE, for phonons which is computationally expensive and require an important number of convergence tests.^13^ Second, materials transport properties are extremely sensitive to synthetic, processing, and operando conditions. For instance, electrical conductivity, σ, can be modified in many orders of magnitude based on composition, carrier concentration, microstructure, or temperature.^14^ This example illustrates that theoretical frameworks need to go beyond stoichiometric single-crystal models in order to provide accurate and useful predictions. Last but not least, model validation through experimental measurements can be extremely complicated due to the lack of well-characterized samples and the inherent uncertainties linked to the experimental measurements.^15,16^

Overcoming these challenges requires a multidisciplinary effort wherein not only more affordable and accurate frameworks are developed but also well-characterized experimental samples are made available. In this work, the lattice thermal conductivity of Cu-based sulvanites will be explored by combining DFT-based simulations, Machine Learning, ML, and experiments. Reducing κ_l_ is usually seen as the most straightforward approach to enhance TE performance, while other properties such as electrical conductivity, σ, and Seebeck coefficient, *S*, are strongly coupled. Additionally, ternary copper chalcogenides Cu_3_MX_4_ (M = V, Nb, Ta; X = S, Se, Te) collectively known as sulvanites have been the subject of different studies due to their promising applications in both thin film photovoltaics and TE technologies.^17,18^ These materials are made of Earth-abundant, nontoxic, and sustainable elements, and they can be synthesized via standard solid-state and solution-based methods. While previous studies have been solely based on either theoretical or experimental results,^19,20^ here, both approaches are connected to provide a clear picture of thermal transport phenomena for these compounds. Integrating both strategies is essential not only to understand previous data misinterpretation but also to accelerate accurate charting of this large family of compounds.

## Methodology

### Computational Details

#### Geometry Optimization

First-principles density functional theory (DFT) calculations were undertaken with the VASP program using projector-augmented wave (PAW) potentials. The exchange-correlation functional proposed by Perdew et al. (PBE) was used to compute the total energies with core electrons described using the potentials proposed by Calderon et al.^21^ A high kinetic energy cutoff of 500 eV and a dense Γ-centered 4 × 4 × 4 Monkhorst-Pack mesh of *k*-points to sample the reciprocal space were used. The wave function was considered converged when the difference between the energy of two consecutive electronic steps was smaller than 1 × 10^–9^ eV. To obtain the optimized conventional unit cell geometry, both the atoms positions and the lattice vectors were fully relaxed until the maximum component of the forces over all atoms was less than 10^–7^ eV Å^–1^, including a supplementary support grid to reduce the noise in the computed forces.

#### Supercell Single-Point Calculations and Force Constants

Interatomic force constants, IFCs, were calculated using the hiPhive package.^22^ This package combines forces from random atomic distortions in supercells with machine learning regression. The forces were extracted from 4 × 4 × 4 supercell (512 atoms) static calculations with the same setup as that used for geometry optimizations. A two-step approach was designed to account for the amplitude of atom distortions’ impact on IFC calculation.^23^ First, small random distortions were generated for all atoms in three supercells, and second- and third-order IFCs were extracted using the hiPhive package. Then, 14 new distorted supercells were created, superimposing normal modes with random phase factors and amplitudes corresponding to 300 K, using the second-order IFCs obtained in the previous step. These physically meaningful distortions accelerate convergence of the IFCs with the number of calculated supercells and increase the accuracy of the extracted force constants. The force constants were determined through multilinear regression to DFT forces utilizing the recursive feature elimination, RFE, algorithm; this method has been demonstrated to require fewer structures to converge than ordinary least-squares regression despite being computationally more expensive.^24^ Furthermore, the RFE reduces parameter count and simplifies models by preserving only significant interaction terms. IFCs were calculated including cutoffs for second-, third-, and fourth-order terms. To ensure transferability across compounds, cutoffs were determined based on coordination shells. For the second-order terms, all interactions within the 4 × 4 × 4 supercell are included; for the third-order terms, we include pairs of atoms that are at a distance lower or equal than the second Cu-M coordination shell; finally, for the fourth-order terms, we include a pair of atoms that are at a distance lower or equal than the first Cu-M coordination shell. These fourth-order terms are included to improve convergency on the RFE algorithm when extracting the second- and third-force constants but are not used later in the calculation of the lattice thermal conductivity.

#### BTE Solver

ShengBTE code is used to calculate the lattice thermal conductivity, κ_l_, through the iterative solution of the BTE, which produces better results than the relaxation time approximation.^25^ Scattering times were computed, including isotopic and three-phonon scattering. Memory demand and the convergence of κ_l_ with the number of *q*-points were balanced using a Gaussian smearing of 0.1 and a dense mesh of 28 × 28 × 28 *q*-points. The effect of grain size on thermal conductivity is discussed based on a decomposition of the contributions to κ_l_ by the phonon mean free path.^26^ This approach has been widely used in the theoretical investigation of nanostructuring effects on thermal transport in thermoelectric materials.^14,27,28^ The value of κ_l_ corresponding to a particular particle size *L* is approximated as the cumulative contributions for all mean free paths up to *L*, effectively subtracting the contributions from mean free paths longer than the particle size. Vacancy point defects have been modeled using the Tamura model wherein an extra scattering rate term is included based on mass variance.^29^ It is worth mentioning that there are more computationally demanding approaches that can describe these systems in a more accurate way such as T-matrix scattering theory.^30,31^ However, it has also been demonstrated that the atomic mass difference plays the key role on the reduction in the lattice thermal conductivity for point defects.^32^ Some previous works have shown how mass difference can be used successfully as the main feature to model the lattice thermal conductivity of systems with vacancies.^33−35^

### Synthesis and Characterization

#### Synthesis and Sintering

Polycrystalline powder of Cu_3_VSe_4_ was synthesized via a solid-state reaction using commercially available powders of Cu (99.7%, Sigma-Aldrich); V (99.9%, Sigma-Aldrich); and Se (99.99%, Sigma-Aldrich). Stoichiometric amounts of these powders were weighted, with the exception of selenium, for which a 1% excess was added. This additional 1% Se was introduced to compensate for its volatilization, ensuring the synthesis of a stoichiometric phase. Subsequently, the reagents were loaded into a fused silica tube, which was then evacuated (<1 × 10^–3^ Torr) and sealed. The reaction mixture was initially heated to 250 °C for 6 h with a heating rate of 1 °C min^–1^ to ensure complete melting of selenium and its reaction with the other elements. The furnace temperature was then raised to 580 °C using a heating rate of 2 °C min^–1^, maintaining this temperature for 72 h, and cooling at the same rate. After cooling, the powder was ground again and placed back into a new vacuum-sealed silica tube, heated up to 540 °C at a rate of 2 °C h^–1^ for 72 h, followed by natural cooling to room temperature.

Finally, a pellet was sintered from the obtaining powder at 600 °C for 15 min under a pressure of 80 MPa using spark plasma sintering (SPS) technique (DR. SINTER LAB Jr sps-212Lx model). The density of the sintered sample was determined using both Archimedes’ method using water as immersion fluid and geometric measurements. The density of the sample exceeded 99% of the theoretical density.

#### Characterization

Phase characterization of the sample was conducted by X-ray diffraction (XRD) employing a Malvern PANalytical Multi-Purpose Diffractometer Empyrean Alpha1. The instrument operates using Cu K_α1_ monochromatic radiation with a wavelength (λ) of 1.54056 Å. Furthermore, neutron powder diffraction (NPD) measurements were performed at room temperature on the D2B beamline at the Institut Laue Langevin (ILL), Grenoble. A radiation source with a wavelength of 1.549 Å was used. NPD data were acquired in high-intensity mode using 2 g of the sample. The structure refinement from the XRD and NPD data was conducted using the FullProf software.^36^ Pseudo-Voigt function was utilized to refine the shape profile, while parameters such as scale factor, manual background, zero-point error, asymmetry, cell parameters, atomic positions, element occupancy, and isotropic atomic displacement parameters were adjusted during the refinement process.

High-resolution electron microscopy (HREM), XEDS (X-ray energy dispersive spectroscopy) analysis, and the exploration of the reciprocal space by means of selected area electron diffraction (SAED) were performed using a JEOL JEM 2100 (double tilt: ±45°) transmission electron microscope (TEM) equipped with a LINK ISIS 300 analyzer. The samples for TEM were prepared by ultrasonic dispersion of the crystals in n-butanol and by depositing drops of the dispersion over a holey carbon-coated 3 mm copper grid.

The morphology and particle size were analyzed by using scanning electron microscopy (SEM) with a JEOL 6400 microscope, which is also equipped with the XEDS analyzer.

Thermal diffusivity (α) measurements were performed on the SPS pellet of Cu_3_VSe_4_ using a Linseis LFA 1000 instrument from 300 to 800 K, using argon as a protective atmosphere. A thin graphite coating was applied to the surface of the pellets to ensure full absorption at the front surface and highest emissivity from the backside. Measurements were taken along the direction of SPS pressing.

Thermal conductivity (κ) was determined using the expression κ = α*C*_p_*d*, where *C*_p_ represents the specific heat, and *d* is the sample density. The specific heat was estimated by using the Dulong–Petit law. The lattice thermal conductivity (κ_l_) was obtained by subtracting the electronic thermal contribution (κ_e_) to the total thermal conductivity. The electronic contribution, κ_e_, was calculated using the Wiedemann–Franz law, κ_e_ = *L*ρ^–1^*T*, were *L* = 2.44 × 10^–8^ W Ω K^–2^ is the Lorenz number, and ρ stands for the electrical resistivity. This last step was measured via the van der Pauw method using a Keithley 2400 source meter unit in a homemade furnace at the specified temperature range. At 823 K, κ_e_ represent less than 1.4% (0.026 W K^–1^ m^–1^) of the total thermal conductivity (1.906 W K^–1^ m^–1^).

## Results

### Cu_3_VSe_4_: Methodology Validation and Comparison with Previous Predictions

**Figure 1:**
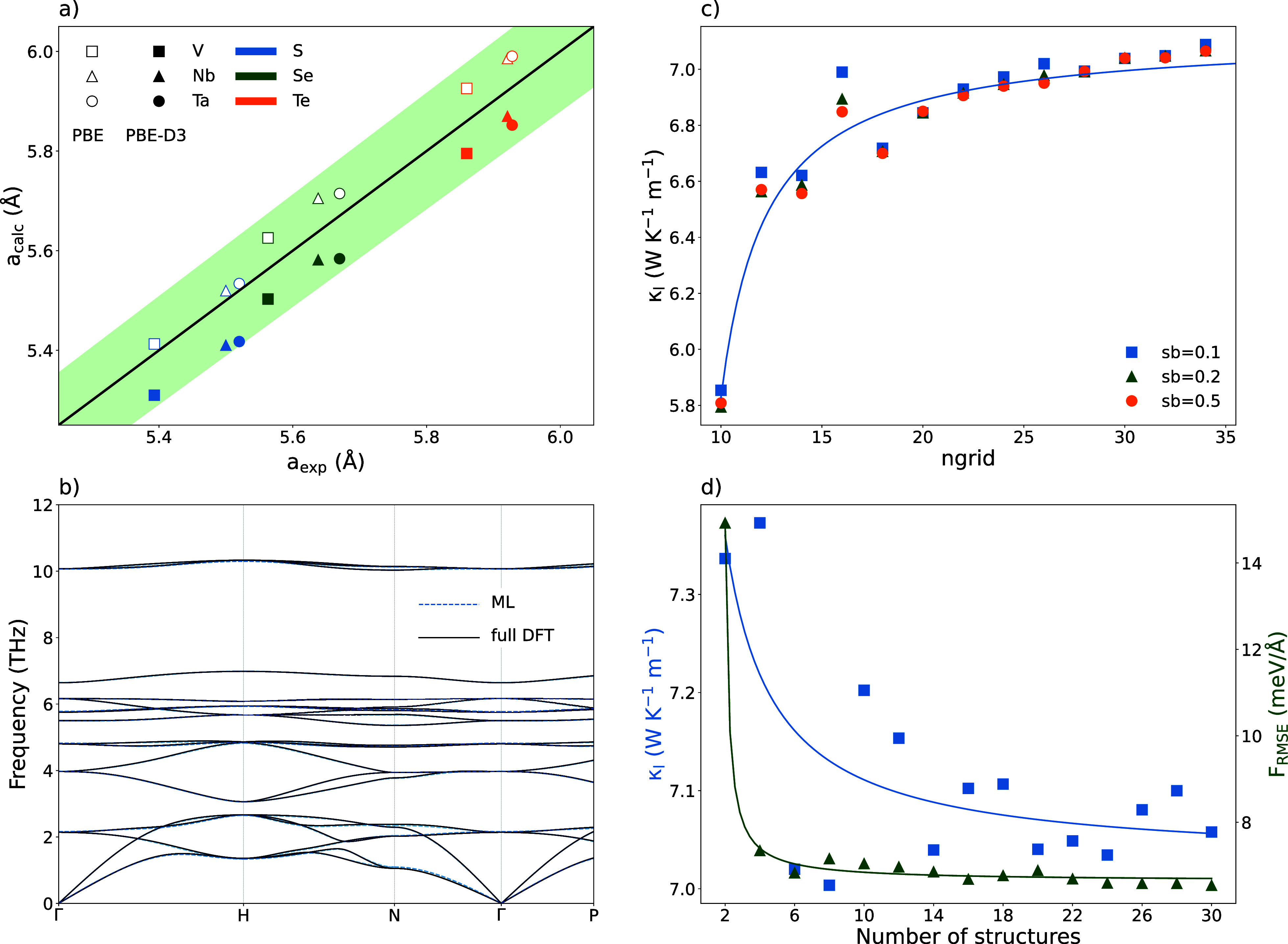
(a) Comparison of optimized cell parameters, *a*_calc_, versus experimental data, *a*_exp_, for Cu_3_MX_4_ compounds. Closed symbols: PBE. Open symbols: PBE-D3. Color code: red, X = S; green, X = Se; blue X = Te; squares: V; triangles: Nb; circles: Ta. The green shaded area represents deviations of ±2% from experiment. (b) Comparison of Cu_3_VSe_4_ dispersion curves obtained from the ML learned force constants (ML) with those from finite difference method via Phonopy (Full-DFT). (c) Computed κ_l_ of Cu_3_VSe_4_ obtained by solving the BTE using ShengBTE as a function of grid density (ngrid) and scalebroad (sb) parameter. (d) Convergence of computed κ_l_ of Cu_3_VSe_4_ (blue) with the number of structures used in the ML algorithm to obtain force constants. On the right axis (green), the evolution of the root-mean-square error on the fitting forces is represented.

Accurate cell parameters are required to compute good phonon properties. Two different functionals, PBE and PBE-D3 (that includes a van der Waals dispersion-energy correction term to the energy^37^) have been used to compute sulvanites lattice parameters. While D3 corrections are usually used to correctly describe layered materials where van der Waals interactions play an important role, previous studies have demonstrated that PBE-D3 can provide a good description of lattice parameters and forces of 3D bulk materials with large anions such as chalcogenides,^23^ pnictides,^38^ and empty skutterudites.^39^Figure 1a shows a comparison of the optimized cell parameters for all Cu_3_MX_4_ compounds calculated with either PBE or PBE-D3 functional and the experimentally available data.^40−48^ The error is never larger than ±2%, but the errors of the PBE-D3 computed cell parameters are larger than those calculated with the PBE functional, which never surpass the ±1%. The sensibly shorter cell parameters computed with the PBE-D3 functional indicates a harder description of the bonds within the solid, and this will lead to higher phonon frequencies and larger κ_l_. Although it has been found that standard GGA functional tend to overestimate cell volumes,^49^ PBE functional results in better agreement with experimental data for sulvanites, specially for sulfides and selenides. However, when tellurides (the largest anion) are considered, both PBE and PBE-D3 deviate similarly from experimental data. We can see that the nature of the chalcogenide anion has the strongest effect on the cell parameter, with tellurides being the compounds with the larger cell volumes and sulfides the smallest. The transition-metal cation, M, also affects the cell parameter but to a much smaller degree: compounds with V have the smallest lattice parameter and those with Nb and Ta the largest, with small differences between these two.

The next step to validate our methodology is to check how the ML potential predicts the second-order force constants. To this end, a comparison of the vibrational dispersion curves for Cu_3_VSe_4_ (Cu_3_VS_4_) obtained from the ML algorithms implemented in the hiPhive package with those obtained with the computationally more expensive finite differences method is presented in Figure 1b (Figure S1), where force constants are obtained from DFT calculations for all symmetry distinct displacement of individual atoms using the Phonopy package. The dispersion curves obtained with both methodologies are virtually identical, showing the equivalence of the two sets of second-order force constants.

**Figure 2:**
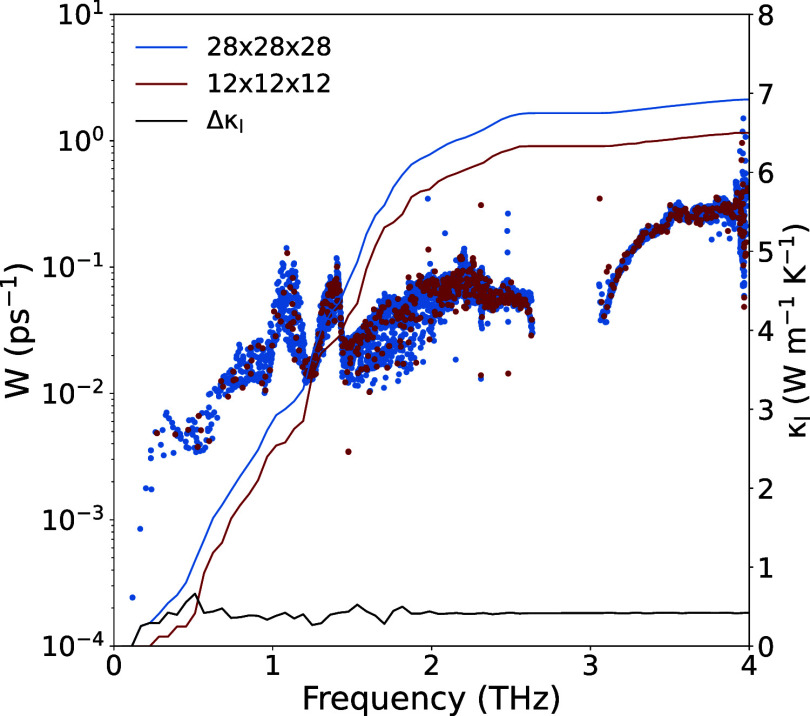
Scattering rates (W in ps^–1^) and cumulative lattice thermal conductivity (κ_l_ in W m^–1^ K^–1^) at 300 K computed with a 12 × 12 × 12 grid of *q*-points (red) and with a denser grid of 28 × 28 × 28 *q*-points (blue) for the Cu_3_VSe_4_ sulvanite.

In Figure 1c, the evolution of the computed κ_*l*_ for Cu_3_VSe_4_ as a function of two important computational parameters is analyzed: the density of the grid of *q*-points and the scalebroad parameter, both settings used in the ShengBTE package to solve the BTE. We can see that for a grid density around 12 × 12 × 12 *q*-points, there seems to be a plateau on the trend of the computed κ_l_. The same tendency has been observed for Cu_3_VS_4_ (Figure S2). This is probably the reason why previous studies have used grid densities in this range.^19,50^ However, if denser grids are used, the computed κ_*l*_ increases, converging to a value of 7.16 W K^–1^ m^–1^ only when a grid of at least 28 × 28 × 28 *q*-points is employed, being independent of the scalebroad parameter. To examine the origin of the failure of less dense grids in the calculation of κ_l_, we have represented in Figure 2 the scattering rates and cumulative lattice thermal conductivity (the κ_l_ calculated including phonons up to the given frequency) for the Cu_3_VSe_4_ sulvanite calculated with two grids of different density: an unconverged 12 × 12 × 12 grid of *q*-points and a denser, converged grid of 28 × 28 × 28 *q*-points. Scattering rates show a much higher resolution graph when the denser grid is employed. In particular, we can distinguish the presence of many contributions to the scattering rates at low frequencies (<1 THz) in the denser grid, that are not present when the lower density grid is employed. When the difference in the accumulated κ_l_ as a function of the vibrational frequency is represented, low frequency contributions make up the main difference in the computed κ_l_ with both grids. In the case of Cu_3_VSe_4_, the difference is less than 0.5 W m^–1^ K^–1^, but for Cu_3_VS_4_, the effect of using a denser grid is more significant, resulting in a difference of 1.52 W m^–1^ K^–1^ (Figure S4).

To end the validation of the computational setup, the convergence of the computed κ_l_ with the number of distorted structures used in the training of the ML fitted force field must be checked. In Figure 1d, the calculated lattice thermal conductivity, κ_l_, is observed to oscillate initially, stabilizing only when the number of distorted structures is increased. Meanwhile, the RMSE of the fitted forces decreases rapidly with the number of structures. When 14 or more distorted structures are used, a κ_l_ around 7.04 W m^–1^ K^–1^ is obtained, while the RMSE of the fitted forces is not significantly reduced by employing more than 14 structures. A similar convergence trend is observed for Cu_3_VS_4_ (Figure S3), demonstrating that the theoretical setup can be effectively applied to the broader family of sulvanite compounds. The κ_l_ convergence observed in Figure 1d highlights the importance of using a sufficiently large number of distorted structures to obtain accurate interatomic force constants and hence reliable thermal transport properties. This means that the fitted force field is converged when 14 distorted structures are used compared to 636 that will be needed to obtain a set of third-order force constants using the classical methodology that does not employ multilinear regression to fit the forces to the force field. Given that the DFT calculation of the forces is the bottleneck of the calculation of the lattice thermal conductivities, the ML approach is an order of magnitude more efficient than the full-DFT approach.

The computed κ_l_ obtained corresponds to an ideal defect-free single crystal. However, real samples are typically polycrystalline, and the grain boundaries introduce defects that allow the scattering of phonons, thus reducing the κ_l_. For instance, Wen et al. synthesized polycrystalline Cu_3_VSe_4_ with an average grain size around 1 μm and a κ_l_ of 3.75 W m^–1^ K^–1^.^51^ Therefore, synthesizing well-characterized, defect-free samples is necessary for direct comparison with the presented model.

### Cu_3_VSe_4_ Synthesis and Characterization

#### Structural Characterization

**Figure 3:**
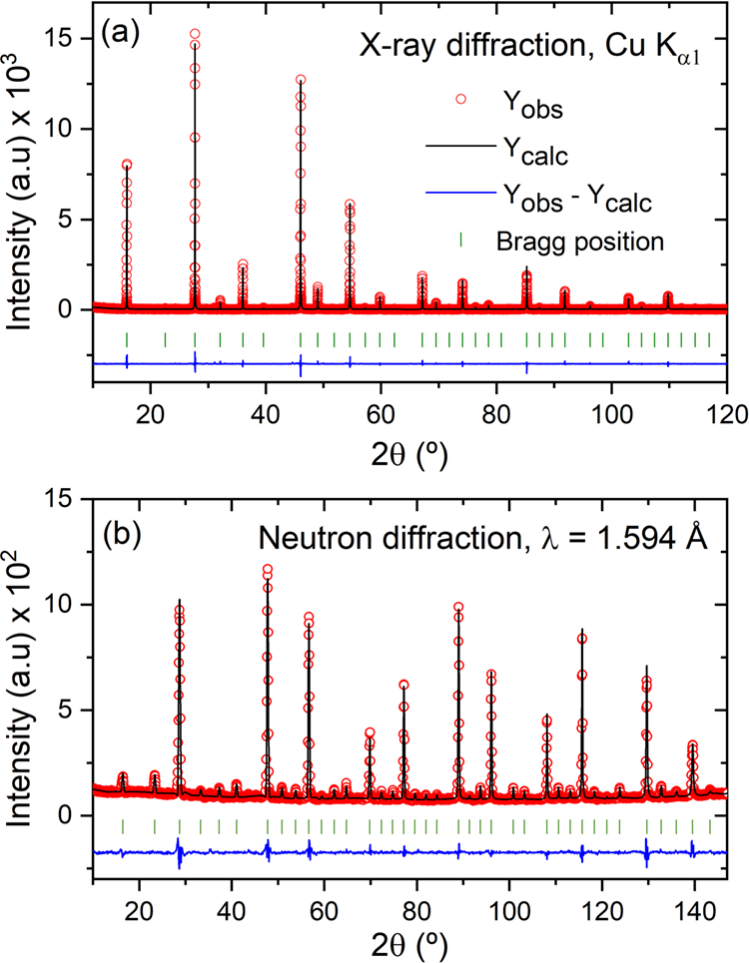
Rietveld refinement of the (a) X-ray diffraction pattern and (b) neutron powder diffraction pattern collected at room temperature for the Cu_3_VSe_4_ sulvanite sample synthesized: the observed pattern (red circle), calculated pattern (black line), the difference diffraction data (blue line), and the Bragg positions (green bars) are shown.

**Figure 4:**
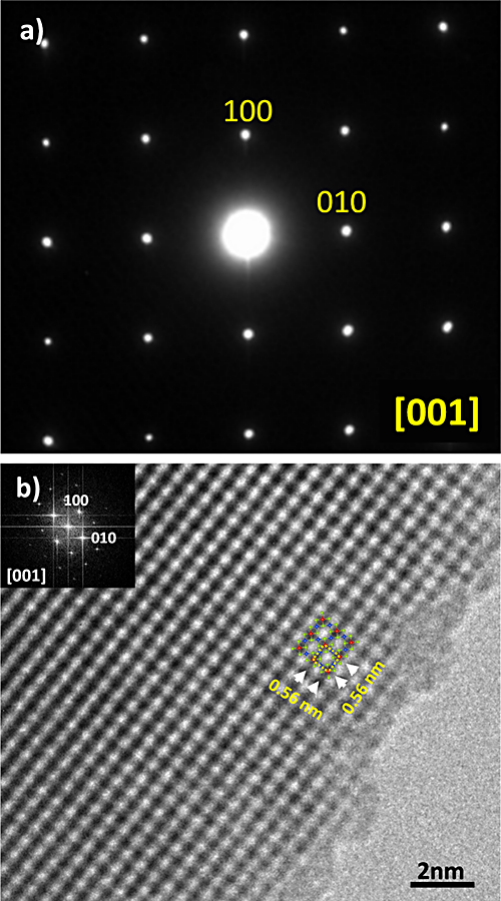
(a) SAED pattern of Cu_3_VSe_4_ along [001] (b) experimental HRTEM micrograph along the [001] zone axis. No streaking or extra spots are evident (see the FFT images in the figure inset). The atoms of the projected cell fit very well with the arrangement of dark spots, where a unit cell of Cu_3_VSe_4_ is marked by the dotted yellow square box.

X-ray diffraction (XRD) and neutron powder diffraction (NPD) measurements of the Cu_3_VSe_4_ phase were carried out to confirm the purity and ascertain the crystallographic structure of the sample at room temperature. Figure 3 shows the Rietveld refinement of the (a) XRD and (b) NPD data. The Cu_3_VSe_4_ diffractograms reveal reflections corresponding to the cubic *P4̅3m* space group, with no indication of a secondary phase. Table 1 summarizes the lattice parameters, atomic positions, isotropic atomic displacement parameters, occupancies, and Rietveld refinement agreement factors. Incorporating vacancies at the different crystallographic positions during refinement has been attempted, resulting in poorer fitting and higher values of the agreement factors compared to maintaining the stoichiometric sample. This result, along with XEDS analyses performed on several microcrystallites, lead to an average composition similar to the nominal one. The selected area electron diffraction (SAED) pattern along the main zone axis [001], displayed in Figure 4a, is in good agreement with the cubic space group *P4̅3m*. The diffraction pattern shows only strong reflections without any streaking or extra reflections, suggesting the absence of extended defects. This is clearly observed in the HRTEM image (Figure 4b), recorded along the same zone axis, where a well-ordered crystal is observed.

**Table 1:** Structural Parameters Were Calculated from the Combined Rietveld Refinement of Cu_3_VSe_4_ XRD and NPD Data

**space group**	*P4̅3m (215)*
*a* (Å)	5.57061(2)
*V* (Å^3^)	172.865(1)

**atomic positions**				
	**Wyckoff positions**	***x***	***y***	***z***
Cu	3*c*	0.5	0	0.5
V	1*b*	0.5	0.5	0.5
Se	4*e*	0.2550(1)	0.2550(1)	0.2550(1)

**isotropic atomic displacement parameters, *U* (Å**^**2**^**)**	
Cu	0.016(5)
V	0.007(8)
Se	0.010(3)

**occupation factor**		**agreement factors (%)**	
Cu	1.0	*R*_wp_	3.85
V	1.0	*R*_exp_	5.53
Se	1.0	*R*_bragg_	1.42

#### SEM

**Figure 5:**
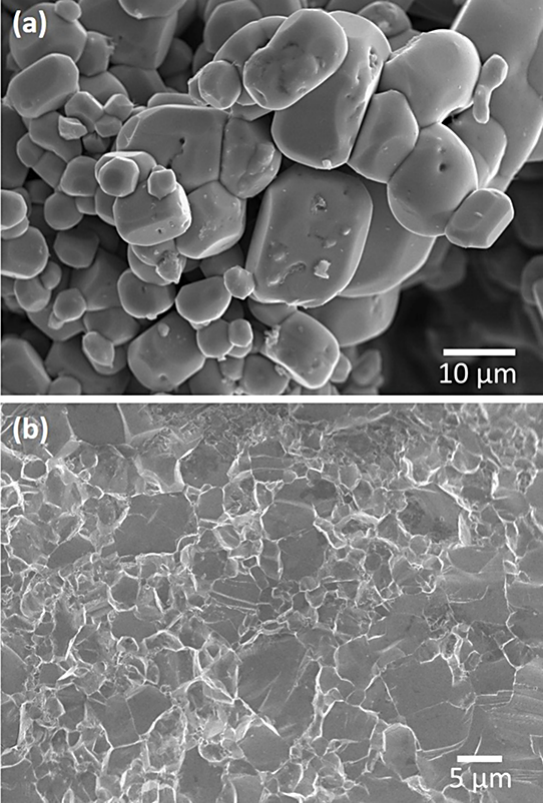
SEM micrographs of Cu_3_VSe_4_ (a) powder and (b) SPS-sintered pellet.

Through SEM imaging, it can be observed that the powdered sample exhibits an agglomerated appearance, with particles of large size, some exceeding 20 μm, due to the extended annealing processes conducted for the synthesis of the phase (Figure 5a). In the case of the pellet prepared by SPS (Figure 5b), effective sintering is observed in the micrograph of the cross section, indicating the elimination of any porosity and confirming proper sintering of the polycrystalline material. The thermoelectric performance of materials typically depends significantly on the density of the pellet. In this case, a relative density of 99% has been achieved.

#### Thermal Conductivity

**Figure 6:**
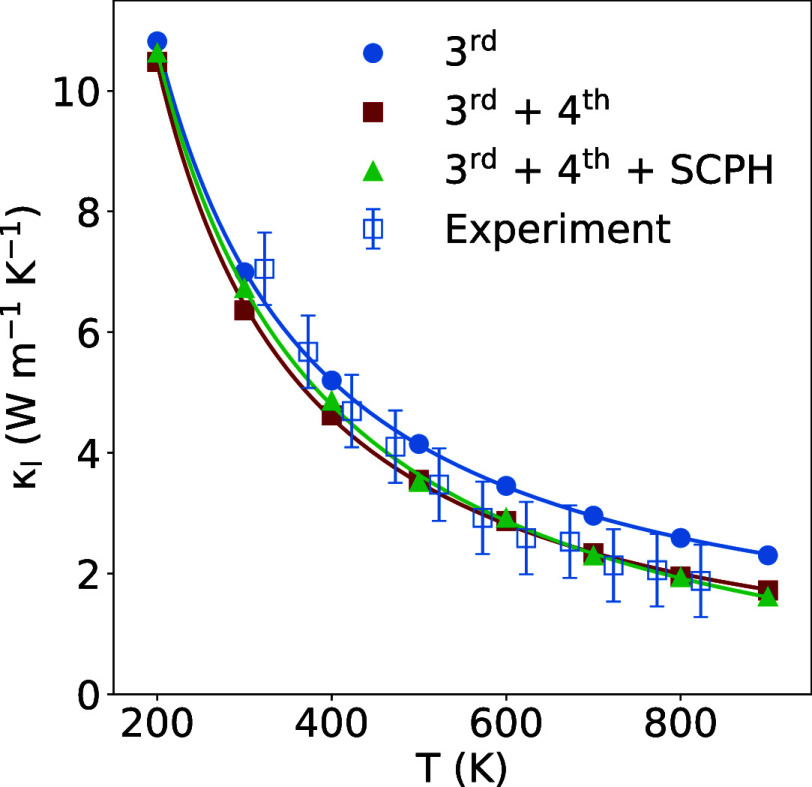
Temperature dependence of the experimentally obtained lattice thermal conductivity (open symbols) compared to the theoretical lattice thermal conductivity (solid line and closed symbols) for the Cu_3_VSe_4_ sulvanite. Lattice thermal conductivity including exclusively third-order scattering processes is depicted in blue. Red and green are used when fourth-order scattering processes and SCPH method are included, respectively. Points were fitted to a *T*^–*n*^ function.

As shown in Figure 6, the experimentally measured (blue empty squares) lattice thermal conductivity of Cu_3_VSe_4_ decreases with increasing temperature, from 7.07 W m^–1^ K^–1^ at 323 K to 1.90 W m^–1^ K^–1^ at 823 K. Computed lattice thermal conductivity values including the third-order scattering process (blue solid circles) closely match experimental measurements across all temperature ranges, with a deviation below the experimental error. However, there is a consistent underestimation of the experimental data, specially at higher temperatures. Although these results can be considered reasonably accurate, particularly for high-throughput screening purposes, methodological improvements have been incorporated into the model, at least for this compound, to better understand its limitations. To improve the accuracy at high temperatures, we incorporated two key improvements: (i) inclusion of the fourth-order scattering processes in the solution of the BTE and (ii) use of the self-consistent phonon (SCPH) method^52^ to account for the renormalization of phonon frequencies with temperature. The fourth-order interatomic force constants have been computed using a cutoff of 5.5 Å,^53,54^ and SCPH has been applied as implemented in hiPhive code.^22,24,55^ As shown in Figure 6, the inclusion of these additional effects brings the computed results into an even closer agreement with the experimental data across all temperatures. Moreover, the SCPH method slightly corrects the underestimation that including fourth-order IFCs produces in the lattice thermal conductivity at room temperature.

**Figure 7:**
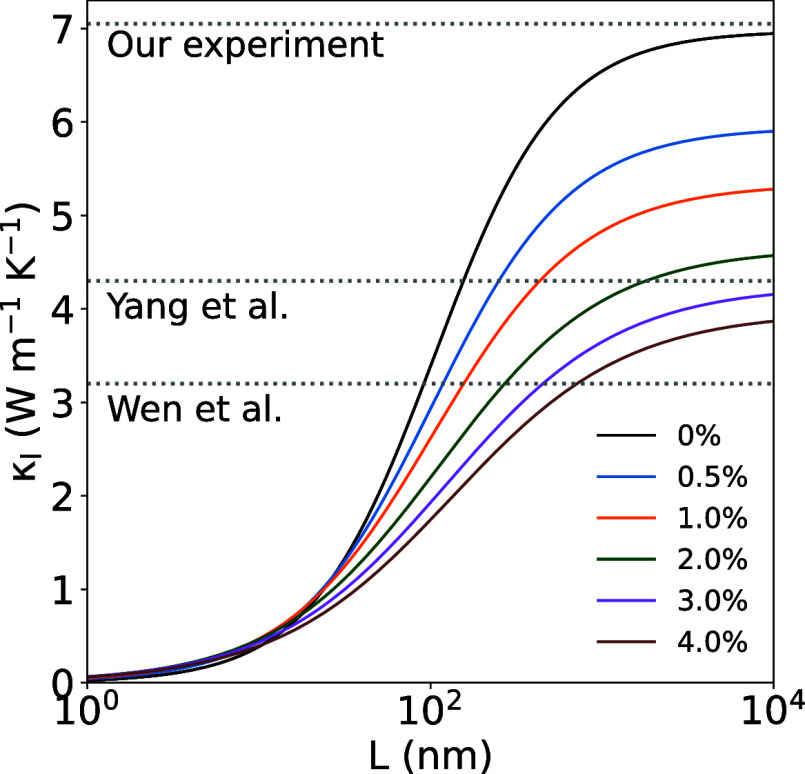
Calculated κ_l_ for different % of Se vacancies as a function of grain size using the Tamura model for Cu_3_VSe_4_. Different experimental values^51,56^ are included with dashed lines.

Previous experimental studies on the thermoelectric properties of this sulvanite reported lower thermal conductivity values compared with those obtained in this work. For example, Wen et al.,^51^ reported an experimental value of 3.7 W m^–1^ K^–1^ for the total thermal conductivity of Cu_3_VSe_4_ at 323 K, which dropped to 1.5 W m^–1^ K^–1^ at 573 K, although they did not distinguish between electronic and lattice contributions. Meanwhile, Yang et al.^56^ reported slightly higher lattice thermal conductivity of 5.79 W m^–1^ K^–1^ at room temperature using a homemade apparatus and 4.27 W m^–1^ K^–1^ at the same temperature using a Laser Flash Analysis instrument. This discrepancy underscores the importance of confirming experimental findings with theoretical calculations. It is manifest that small variations in vacancy concentration or differences in particle size or morphology can significantly influence phonon transport, thereby affecting thermal conductivity. Unfortunately, none of the previous studies provided experimental data on the occupancy of the constituent elements, which could offer insights into these discrepancies. The cumulative thermal conductivity has been calculated using the Tamura^29^ model to explore the simultaneous effect of grain size and Se vacancies on κ_l_ (Figure 7). Se vacancies were included in the model as an example of point defects due to the volatility of Se. This type of analysis can be helpful for (i) designing samples with tailored thermal conductivity and (ii) understanding the origin of the thermal conductivity in synthesized samples. For instance, the samples synthesized by Wen et al.^51^ presents an experimental value of 3.7 W m^–1^ K^–1^, and based on the reported SEM images, the average grain size is around 1 μm. Such a low value of κ_l_ is not compatible with a defect-free polycrystalline sample of that average size based on our model, implying the presence of other defects. To reproduce the experimental value, around 3–4% Se vacancies would be required. We can see that the presence of Se vacancies at relatively low concentrations of 1–2% result in an important reduction of κ_l_. These results indicate that a fair comparison of experimental data and theoretical calculation requires a detailed characterization of the samples, in terms of both particle size and composition.

Although there are many theoretical works that have examined the thermodynamic, optical, and electronic properties of the sulvanite compounds, only a handful of papers have focused on the calculation of their thermoelectric properties. Hong et al.^57^ examined these properties on Cu_3_MX_4_ (M = V, Nb, Ta; X = Se, Te), including κ_l_ and *zT* and related the high *zT* values obtained to the electronic band structure near the valence band maximum. However, the use of the Slack approximation to calculate κ_l_ limits the accuracy of their results. Liu et al.^50^ used compressive sensing lattice dynamics to obtain the anharmonic force constants from standard DFT calculations on Cu_3_VX_4_ (X = S, Se, and Te). The approach used is comparable to the one used in this work; however, they have used a 11 × 11 × 11 grid of *q* points to solve the Boltzmann Transport Equation, BTE, that, as we have shown, is far from being converged. More recently, Haque^19^ studied the full series of compounds Cu_3_MX_4_ (M = V, Nb, Ta; X = S, Se, Te) and found significant discrepancies with previous studies. The authors used the finite displacement method as implemented in Phonopy with a small 2 × 2 × 2 supercell. The phonon Boltzmann transport equation is solved to obtain the phonon structure and the thermoelectric properties with a 12 × 12 × 12 grid of *q* points that is far from being converged. Although in the Supporting Information of this study the vibrational density of states has been decomposed into the different atoms contribution, no analysis relating the variation of κ_l_ to the chemical composition is performed. Yang et al.^56^ also performed some calculations on Cu_3_VSe_4_ and Cu_3_NbSe_4_. They used a 2 × 2 × 2 supercell and Phonopy to obtain the harmonic force constants, but inconsistent details are given about how the third-order force constants were calculated. No details were offered on the setup of the calculations to solve the BTE and obtain the κ_l_. The lower κ_l_ in Cu_3_NbSe_4_ compared to that in Cu_3_VSe_4_ is interpreted in terms of the longer Cu-Se distances in Cu_3_NbSe_4_ and the change in the atomic mass of the TM atom.

Although discrepancies between theory and experiments are common in predicting lattice thermal conductivity, a double error cancellation has masked these differences in the case of sulvanites like Cu_3_VSe_4_. Previous theoretical predictions reported κ_l_ values for Cu_3_VSe_4_ ranging from 3.5 to 5.9 W m^–1^ K^–1^, primarily due to the use of low-density grid.^19,50,56^ Meanwhile, experimental values were also in a similar range (3.2 to 5.8 W m^–1^ K^–1^) but did not account for the impact of grain boundaries and point defects as was discussed before.^51,56^

### High-Throughput Screening of κ_l_ for Cu-Based Sulvanites

**Figure 8:**
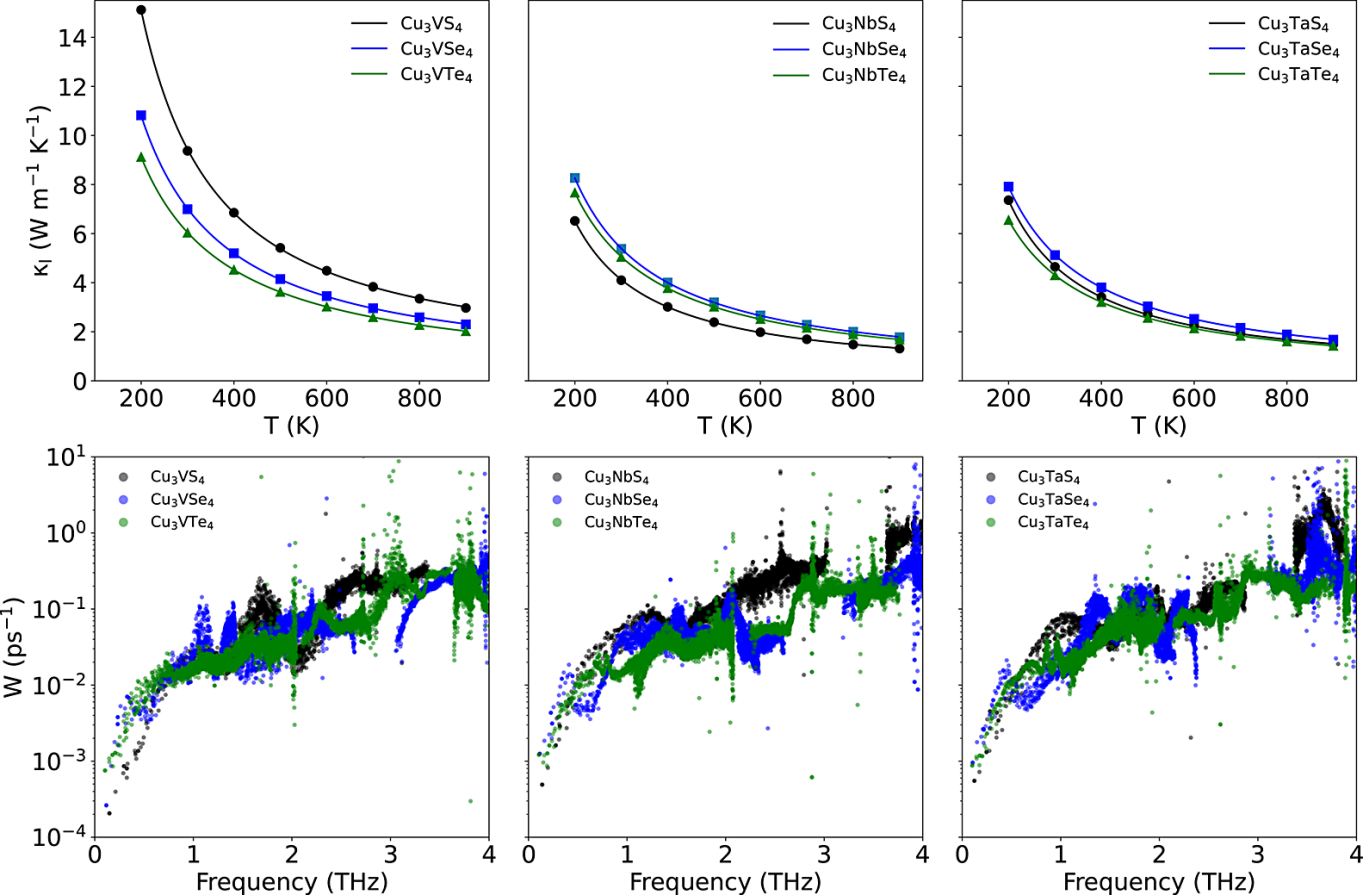
Calculated temperature variation of κ_l_ (top) and scattering rates vs mode frequencies (bottom) at 300 K for the different Cu-based sulvanites studied in this work.

Figure 8 (top) shows the calculated temperature variation for κ_l_ for all Cu_3_MX_4_ sulvanites. The computed κ_l_ follows a T^–1^ trend as expected, and there is a neat effect of the chalcogen on the Cu_3_VX_4_ materials and a considerable reduction on κ_l_ when V is replaced by either Nb or Ta, but the chalcogen effect is less defined in these cases. To rationalize the effects that the chemical composition has on the transport properties of the Cu_3_MX_4_ materials, we will start by examining the effect of the chalcogen on the Cu_3_VX_4_ group of materials. The phonon dispersion diagrams presented in Figure S5 show that the vibrational frequencies of Cu_3_VX_4_ (X = S, Se, and Te) and, in particular, those associated with lower optical modes are considerably reduced when going from S to Se and finally to Te. These dispersion diagrams show how the lower energy optical modes that mix with the acoustic modes are sensibly flattened when S is substituted by Se and even further when Se is substituted by Te. The vibrational structure of the Cu_3_VX_4_ sulvanites, their vibrational density of states, and the cumulative effect on the computed κ_l_ presented in Figure S6 allow to further understand the effect of the chalcogen substitution. While the low-energy vibrational modes are mainly contributed by Cu on Cu_3_VS_4_, the participation of the chalcogen increases in Cu_3_VSe_4_ and is the main contribution in Cu_3_VTe_4_. The discussed flattening of the dispersion curves is the result of an avoided-crossing that takes place between the first optical mode (at ∼2 THz at the Γ point) and the acoustic modes that converge at the Γ point, which are the modes with the higher group velocities. As a consequence, the slopes of the dispersion curves of these acoustic modes are reduced for the heavier chalcogen derivatives, and the group velocities decrease. Group velocities distribution, presented in Figure S7 for the Cu_3_VX_4_ sulvanites, show that these are higher for Cu_3_VS_4_ than for Cu_3_VSe_4_ that, in turns, shows higher group velocities than Cu_3_VTe_4_. Larger group velocities imply better heat transport properties, and thus, these group velocities are aligned with the observed trend in the computed κ_l_ of these materials. Moreover, the scattering rates for the Se- and Te-derivatives, see Figure 8 (bottom), are clearly higher at low frequencies as a result of the low-lying optical modes introduced by these heavier chalcogens, thus again in line with the lower κ_l_ computed for these compounds.

Computed κ_l_ for Cu_3_NbX_4_ and Cu_3_TaX_4_ sulvanites are about half those calculated for the Cu_3_VX_4_ set, showing that there is a large effect of the transition-metal atom on the thermal conductivity (see Figure 8, top). The behavior of κ_l_ with chalcogen substitution is, however, clearly different and not uniform. Surprisingly, the Cu_3_NbS_4_ sulvanite shows the lower thermal conductivity, and there is almost no difference between the computed κ_l_ of the remaining materials. To rationalize this behavior, we can start by looking at the phonon dispersion diagrams of these materials, as presented in Figure S5. The substitution of the lighter V atom by the heavier Nb or Ta transition-metal atoms reduces the vibrational frequencies of the high-energy optical modes, mainly those that appear above 8 THz that are associated to the TM–X bond vibration according to the projected phonon dispersion density of states of Figure S6. Contrarily to the V atom, both Nb and Ta also show contributions to the low-lying (<3 THz) optical modes and, thus, the TM–X vibration also interacts with the acoustic modes (Figure S6). Comparison of the scattering rates (Figure 8) shows that those of the Cu_3_NbX_4_ and Cu_3_TaX_4_ sulvanites are nearly an order of magnitude larger (note the log scale on the *y*-axis) than those of the Cu_3_VX_4_, indicating the high effect of the heavier TM atoms on the thermal transport properties of those compounds. The group velocities distribution (Figure S7) follow the same trend with the chalcogen: they are larger for S and decrease for the heavier chalcogens Se and Te. However, except for Cu_3_NbS_4_, the effect of the chalcogen is minimal showing that now the scattering rates, much larger due to the heavier mass of the TM atoms Nb and Ta, dominate the anharmonic properties of these compounds and, thus, the behavior of κ_l_.

## Conclusions

Accelerated materials discovery requires not only robust and efficient high-throughput theoretical frameworks but also experimental validation. In this work, the thermal conductivity of a large family of thermoelectric materials, Cu-based sulvanites, has been explored. DFT calculations and the solution of the BTE for phonons were combined with ML-regression techniques for the accurate prediction of κ_l_. The comparison with previous reports demonstrates the importance of systematically converging all parameters involved in the workflow. It is highlighted that a dense *q*-point grid projected on the Brillouin zone is essential for a good description of thermal transport properties, using Cu_3_VSe_4_ as a critical example. The presented high-throughput framework has been validated by synthesizing and characterizing defect-free polycrystalline Cu_3_VSe_4_. Experimental and theoretical values for κ_l_ are in very good agreement over a wide range of studied temperatures. In combination with previous reported works, the obtained large grain sizes with the absence of extended defects demonstrate the impact of grain boundaries and defects on the lattice thermal conductivity of Cu-based sulvanites. Finally, κ_l_ has been computed for this whole family of materials. The role of the anion and the transition-metal cation has been rationalized based on their dispersion curves, group velocities, and scattering rates. Some of these compounds, such as Cu_3_NbS_4_, present lattice thermal conductivity below 2 W m^–1^ K^–1^ above 600 K, opening the door to further optimization of their thermal transport properties for their use in thermoelectric devices.

## Data Availability

Data are available at the ZENODO repository (doi:10.5281/zenodo.13625573).

## Supporting Information Available

Graphics describing theoretical setup validation for Cu_3_VS_4_; computed phonon dispersion curves, phonon density of states, cumulative κ_l_, scattering rates, and group velocities for all different Cu-based sulvanites studied in this work (PDF)
