# Supplementary material for: Challenges Reconciling Theory and Experiments in the Prediction of Lattice Thermal Conductivity: The Case of Cu-Based Sulvanites
Source: Chem Mater. 2024 Sep 4;36(18):8704–13. doi: 10.1021/acs.chemmater.4c01343 (PMC11428157; doi:10.1021/acs.chemmater.4c01343)
Supplement: Supplementary file 1 — cm4c01343_si_001.pdf [file cm4c01343_si_001.pdf]

Supporting Information: Challenges reconciling theory and experiments in the prediction of lattice thermal conductivity: the case of Cu-based sulvanites

Irene Caro-Campos,[†,∥] Marta María González-Barrios,[‡,∥] Oscar J. Dura,[¶] Erik Fransson,[§] Jose J. Plata,[†] David Ávila,[‡] Javier Fdez. Sanz,[†] Jesús Prado-Gonjal,[‡] and Antonio M. Márquez[*,†]

†*Departamento de Química Física, Facultad de Química, Universidad de Sevilla, E-41012, Seville, Spain*

‡*Departamento de Química Inorgánica, Universidad Complutense de Madrid, E-28040, Madrid, Spain*

¶*Departamento de Física Aplicada, Universidad de Castilla-La Mancha, E-13071, Ciudad Real, Spain*

§*Department of Physics, Chalmers University of Technology, SE-41296 Gothenburg, Sweden*

∥*These two authors contributed equally*

E-mail: marquez@us.es

Abstract

The exploration of large chemical spaces in search of new thermoelectric materials requires the integration of experiments, theory, simulations, and data science. The development of high-throughput strategies that combine DFT calculations with machine

learning has emerged as a powerful approach for discovering new materials. However, experimental validation is crucial to confirm the accuracy of these workflows. This validation becomes especially important in understanding the transport properties that govern the thermoelectric performance of materials since they are highly influenced by synthetic, processing, and operating conditions. In this work, we explore the thermal conductivity of Cu-based sulvanites using a combination of theoretical and experimental methods. Previous discrepancies and significant variations in reported data for Cu_3VS_4 and Cu_3VSe_4 are explained using the Boltzmann Transport Equation for phonons and by synthesizing well-characterized defect-free samples. The use of machine learning approaches for extracting high-order force constants opens doors to charting the lattice thermal conductivity across the entire Cu-based sulvanite family—finding not only materials with κ_l values below 2 W m^{-1} K^{-1} at moderate temperatures but also rationalizing their thermal transport properties based on chemical composition.

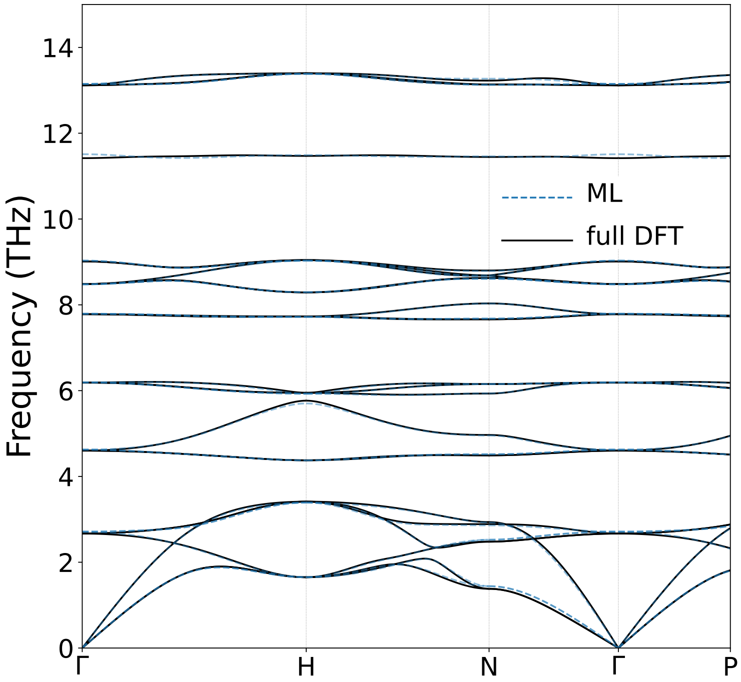

Figure S1: Comparison of Cu_3VS_4 dispersion curves obtained from the ML learned potential (ML) with those from the finite differences method via Phonopy (Full-DFT).

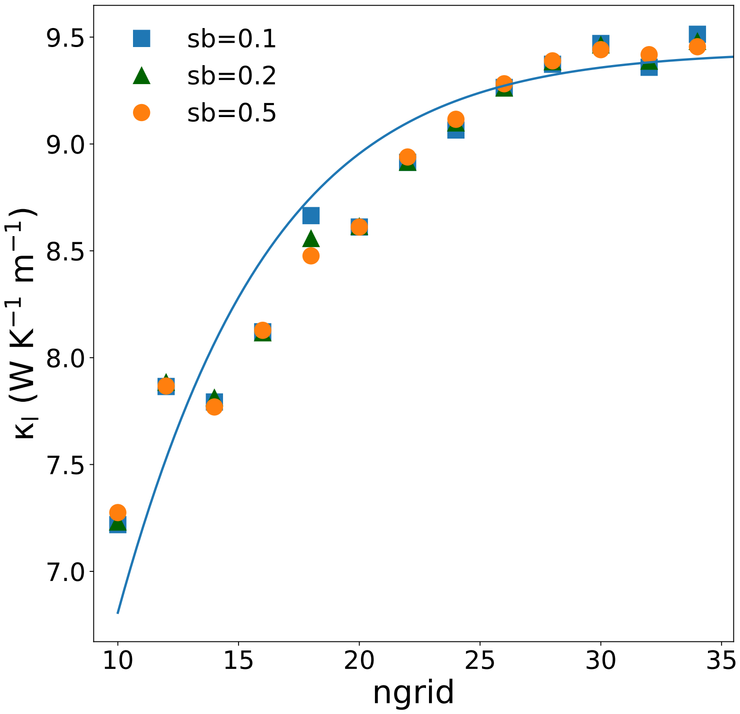

Figure S2: Convergence of computed κ_l for Cu_3VS_4 obtained solving the BTE by using the ShengBTE code as a function of grid density and scalebroad (sb) parameter.

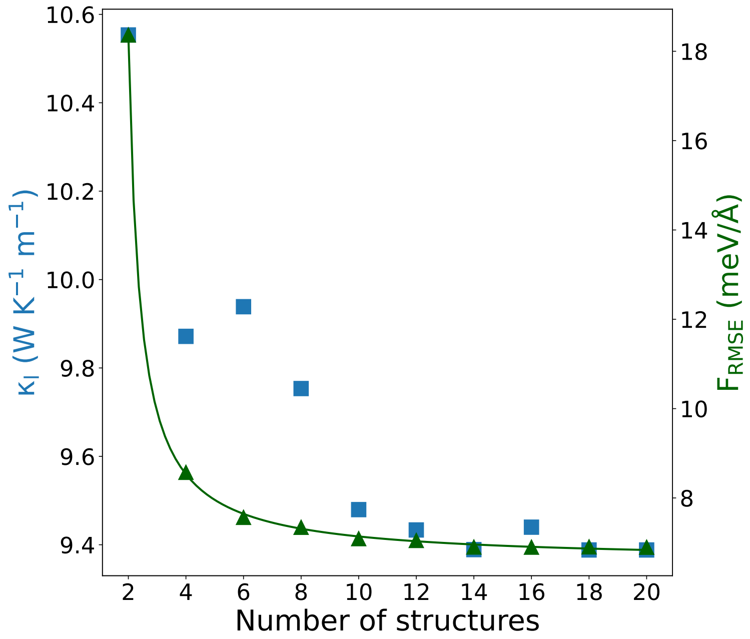

Figure S3: Convergence of computed κ_l for Cu_3VS_4 (blue) with the number of structures used in the ML algorithm to obtain the force constants. On the right axis (green), the evolution of the root-mean-square error of the fitting forces is represented.

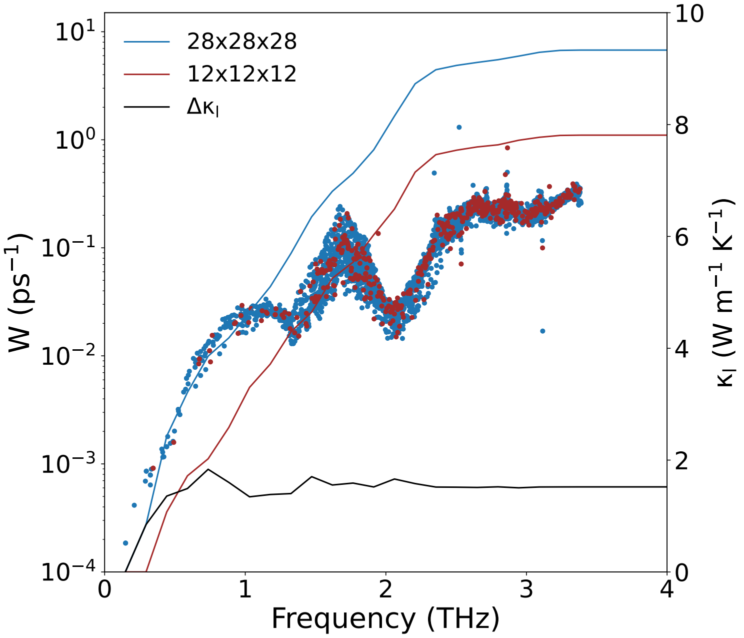

Figure S4: Scattering rates (W in ps^{-1}) and cummulative lattice thermal conductivity (κ_l in W m^{-1} K^{-1}) at 300 K computed with a $12 \times 12 \times 12$ grid of **q**-points (red) and with a denser grid of $28 \times 28 \times 28$ **q**-points (blue).

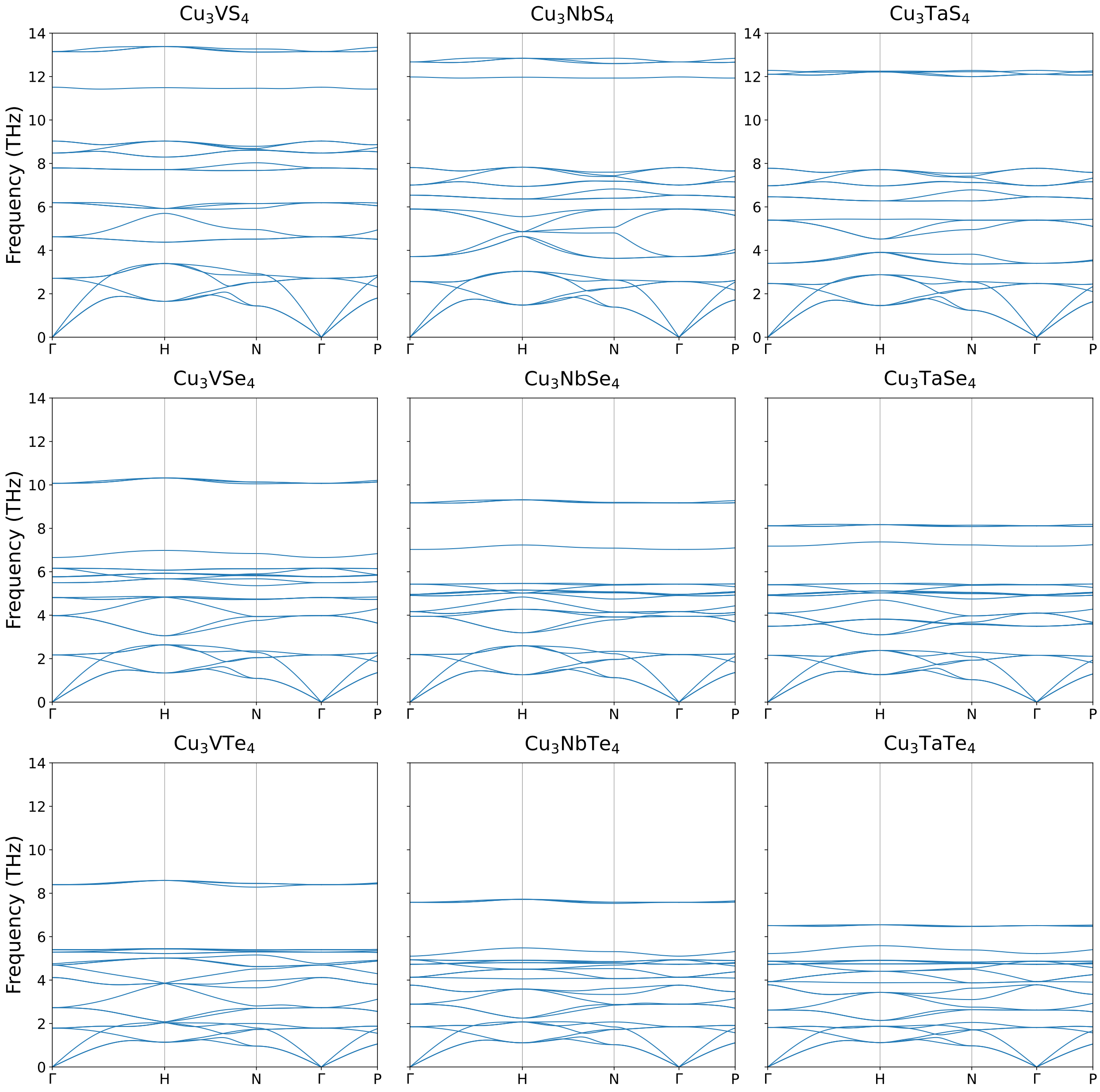

Figure S5: Dispersion diagrams for all Cu_3MX_4 (M=V, Nb, Ta; X=S, Se, Te) compounds.

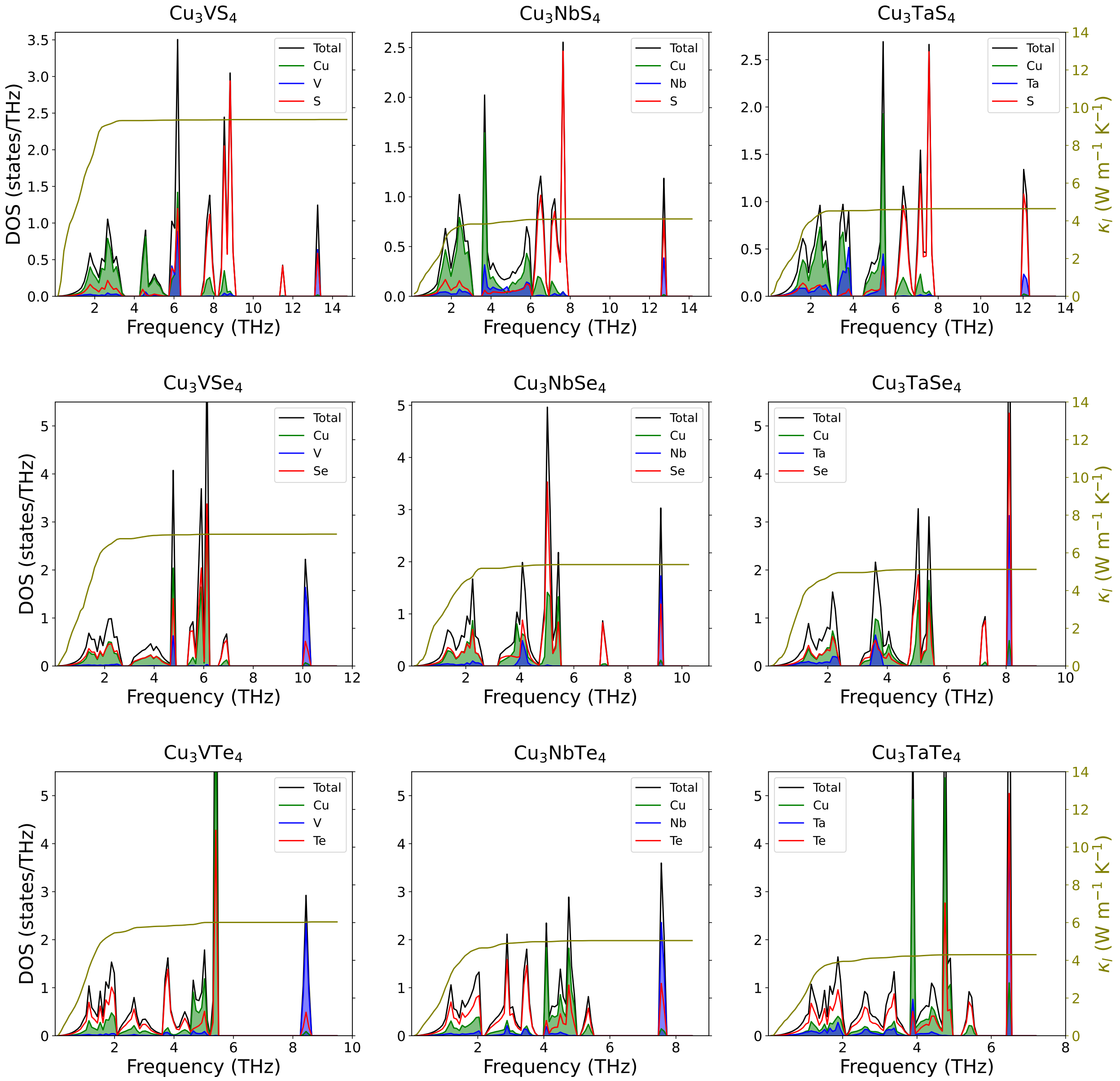

Figure S6: Vibrational density of states and cummulative κ_l at 300 K for al Cu_3MX_4 (M=V, Nb, Ta; X=S, Se, Te) compounds.

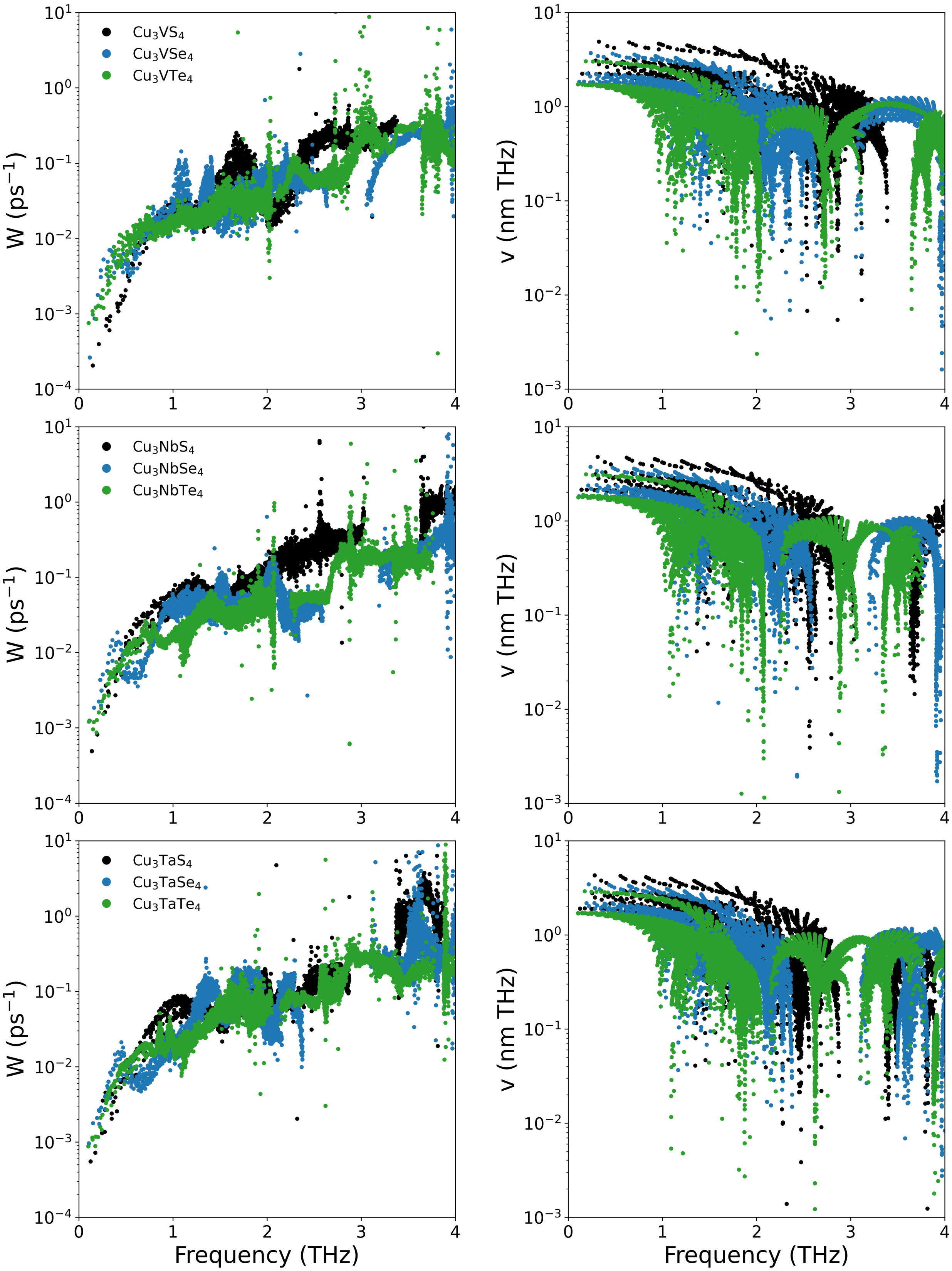

Figure S7: Scattering rates and group velocities at 300 K for all Cu_3MX_4 (M=V, Nb, Ta; X=S, Se, Te) compounds.
